# Evaluation of a central venous catheter tip placement for superior vena cava–subclavian central venous catheterization using a premeasured length: A retrospective study

**DOI:** 10.1097/MD.0000000000009600

**Published:** 2018-01-12

**Authors:** Hyun-Jung Kwon, Young-Il Jeong, In-Gu Jun, Young-Jin Moon, Yu-Mi Lee

**Affiliations:** Department of Anesthesiology and Pain Medicine, Asan Medical Center, University of Ulsan College of Medicine, Seoul, Republic of Korea.

**Keywords:** carina, chest radiograph, placement, subclavian central venous catheterization, superior vena cava

## Abstract

Subclavian central venous catheterization is a common procedure for which misplacement of the central venous catheter (CVC) is a frequent complication that can potentially be fatal. The carina is located in the mid-zone of the superior vena cava (SVC) and is considered a reliable landmark for CVC placement in chest radiographs. The C-length, defined as the distance from the edge of the right transverse process of the first thoracic spine to the carina, can be measured in posteroanterior chest radiographs using a picture archiving and communication system. To evaluate the placement of the tip of the CVC in subclavian central venous catheterizations using the C-length, we reviewed the medical records and chest radiographs of 122 adult patients in whom CVC catheterization was performed (from January 2012 to December 2014) via the right subclavian vein using the C-length. The tips of all subclavian CVCs were placed in the SVC using the C-length. No subclavian CVC entered the right atrium. Tip placement was not affected by demographic characteristics such as age, sex, height, weight, and body mass index. The evidence indicates that the C-length on chest radiographs can be used to determine the available insertion length and place the right subclavian CVC tip into the SVC.

## 1 Introduction

Central venous catheterization is a common procedure in patients who are critically ill or undergoing major surgery, but central venous catheter (CVC) misplacement is a frequent complication of this procedure and can potentially be fatal.^[1,2]^ Several guidelines recommend that the tip of a CVC be located in the superior vena cava (SVC).^[1–3]^ To minimize the risk of cardiac tamponade, it has been suggested that the tip of the CVC should be located above the cephalic limit of the pericardial reflection, not merely above the junction of the SVC and right atrium (SVC/RA).^[1]^ Although electrocardiography (ECG), ultrasound guidance, or the guidelines of the UK's National Institute for Health and Clinical Excellence may be used to assess the location of the CVC, these methods are not routinely available in the operating room.^[4–6]^ Several techniques^[7,8]^ or landmarks^[9,10]^ have been proposed for positioning the CVC to an adequate depth in adults. Chest radiographs are considered a practical and reliable method of confirming the placement of the tip of the CVC in the SVC, and the carina, which is located in the mid-zone of the SVC, is considered a safe and accurate landmark for tip placement.^[9–11]^ Anatomical landmarks play essential roles in guiding medical procedures. Regardless of the age, sex, height, or weight of the patient or the skill of the physician, they are always present and easy to find, and results obtained with their use should be accurate and reproducible.

**Figure 1:**
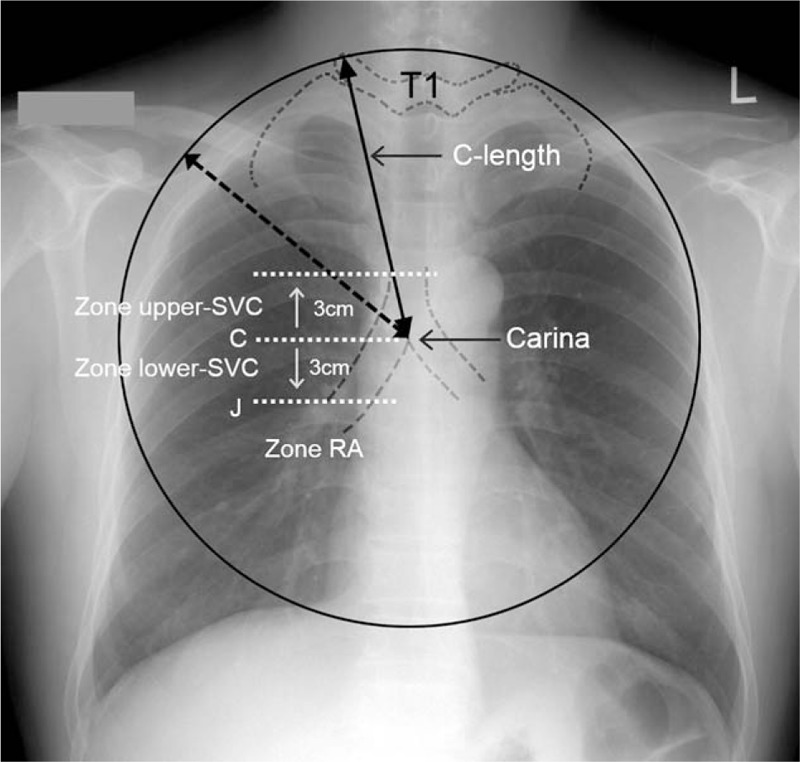
Diagram of the C-length and the zone of central venous catheter placement. C-length (straight arrow line) was defined as the distance from the edge of the right transverse process of the first thoracic spine (T1) to the carina. C = level of the carina, J = SVC/RA junction, lower SVC = between the carina and 3 cm below it, RA (right atrium) = below the J, SVC = superior vena cava, upper SVC = between the carina and 3 cm above it. Adapted from Lee and Lee.^[12]^

In a previous study, 2 landmarks in chest radiographs were used for right internal jugular vein catheterization to place the tip of the CVC in the SVC; these were the edge of the right transverse process of the first thoracic spine (T1) and the carina, with the distance between these 2 landmarks termed the C-length (Fig. 1).^[12]^ It is difficult to place CVC tips above the RA in all cases using conventional techniques.^[13]^ In this study, we evaluated whether the C-length could be used as an available insertion length for right subclavian vein (SCV) catheterization in placing the tip of the CVC in the SVC.

## 2 Methods

### 2.1 Patients

This study was approved by the Institutional Review Board of Asan Medical Center (no. 2017-0391). We retrospectively collected adult patients in whom CVC catheterization was performed via the right SCV using the C-length in operating rooms in Asan Medical Center, Seoul, Republic of Korea, between January 2012 and December 2014. A total of 122 adult patients who underwent major abdominal procedures and surgical neurological procedures were included in the present study. Medical records were reviewed for demographic characteristics, including sex, age, height, weight, and body mass index (BMI). To evaluate the tip placement of the CVC in SCV catheterizations using the C-length, chest radiographs of the 122 enrolled patients were reviewed.

### 2.2 Subclavian vein catheterization

The C-length, defined as the distance from the edge of the right transverse process of the first thoracic spine to the carina, was measured in preoperative posteroanterior chest radiographs using a picture archiving and communication system (PACS)^[14]^ (Fig. 1).^[12]^ After the induction of general anesthesia, the patients were placed in a slight Trendelenburg position with the head turned to the left. The infraclavicular approach was used for right SCV catheterization in all 122 patients. After antiseptic preparation and draping, a 20 cm, triple-lumen CVC (ARROWgard Blue; Arrow, Reading, PA) was inserted over a guidewire using a modified Seldinger technique. All subclavian CVCs were inserted into their respective C-lengths. Afterwards, each patient was returned to the supine position and his/her head and neck were placed in the neutral position. The placement of the tip of the CVC was confirmed by chest radiography after catheterization. There were no complications such as arterial puncture or pneumothorax.

### 2.3 CVC tip placement

CVC tip placement was divided into three zones, that is, the upper SVC, lower SVC, and RA. The upper SVC zone was defined as the zone between the carina and 3 cm above it (Fig. 1).^[12]^ The lower SVC zone was defined as the zone between the carina and 3 cm below it, and the RA zone was defined as the zone beginning 3 cm below the carina (Fig. 1).^[12]^ The 3 cm below the carina was determined to be on the lower border of the SVC, because the carina is roughly 3 to 4 cm higher than the SVC/RA junction in adults^[15,16]^ and the SVC/RA junction cannot be identified with certainty on chest radiographs.^[9]^ The perpendicular distance from subclavian CVC tip to the level of the carina was measured.^[12]^ The upper SVC zone was divided into 2 subzones for ease of comparison of demographic characteristics, including sex, age, height, weight, and BMI. The upper SVC (A) was defined as the subzone between 3 and 1.5 cm above the carina, and the upper SVC (B) was defined as the subzone between the carina and 1.5 cm above it.

### 2.4 Statistical analysis

Data are expressed as the number of patients (ratios or percentages), and means ± standard deviations as appropriate. Data were compared using the *t* test or the Mann–Whitney *U* test for continuous variables, or the *χ*^2^ test for categorical variables. We analyzed the normality of our data by tests of normality, including the Kolmogorov–Smirnov and Shapiro–Wilk tests. The lower, but not the upper, SVC data followed a normal distribution. Therefore, we used the Mann–Whitney *U* test for comparing the upper and the lower SVC data. All statistical analyses were performed using the SPSS statistical package, version 20.0 (SPSS, Inc., Chicago, IL) for Windows. A *P*-value < .05 was considered statistically significant.

## 3 Results

**Figure 2:**
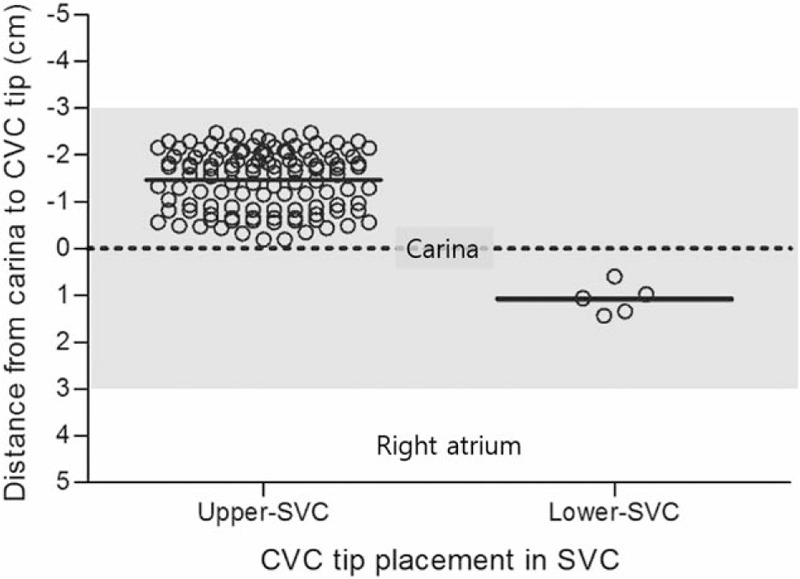
Scatter plot of tip placement of subclavian CVCs using the C-length from a chest radiograph. CVC = central venous catheter, lower SVC = between the carina and 3 cm below it, SVC = superior vena cava, upper SVC = between the carina and 3 cm above it. The gray shade indicates the zone of SVC.

**Figure 3:**
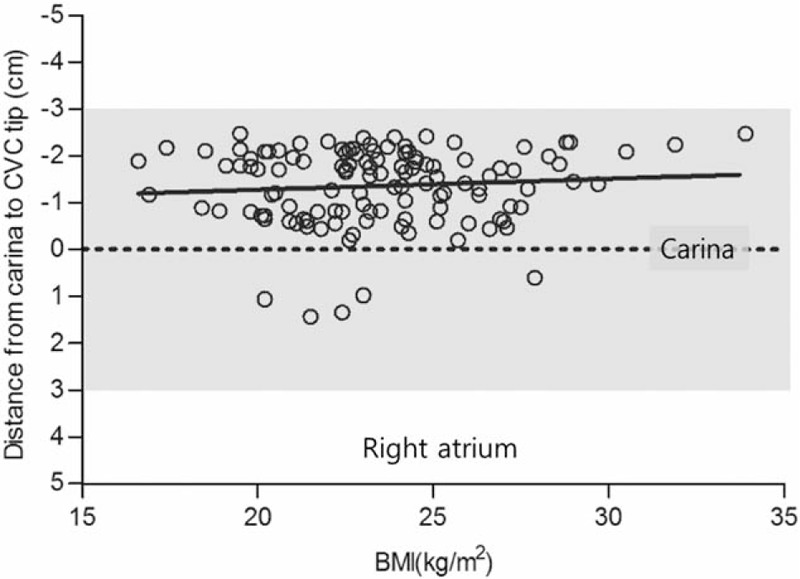
Relationship between tip placement of subclavian CVCs and BMI on chest radiographs. BMI = body mass index, CVC = central venous catheter. The gray shade indicates the zone of the superior vena cava.

Patient characteristics are presented in Table 1. Heights were in the range of 145.0 to 204.0 cm, weights were 38.5 to 106.0 kg, BMIs were 16.6 to 33.9 kg/m^2^, and subclavian CVC insertion distances from the skin puncture site were 9.4 to 16.5 cm (Table 1). In all, 117 tips of CVCs were placed in the upper SVC and 5 were placed in the lower SVC (*P* < .001) (Table 2, Fig. 2). The tips of all subclavian CVCs using the C-length were placed in the SVC and there was no subclavian CVC that entered the RA (Fig. 2). The distance from the tip to carina was −1.47 ± 0.64 cm (−2.48 to −0.20) above the carina in the upper SVC and was 1.07 ± 0.32 cm (0.6–1.42) below the carina in the lower SVC (*P* < .001) (Table 2). The C-length was not different between the upper and lower SVC (*P* = .159). Among patients in whom the subclavian tip of the CVC was placed in the SVC, there were no statistically significant differences in age, sex, height, weight, or BMI between patients in whom the tip was placed in the upper SVC (A), upper SVC (B), and lower SVC (Table 3). There were also no statistically significant differences in CVC length or insertion range of the among those who had placement in the upper SVC (A), upper SVC (B), and lower SVC in (Table 3). There were no statistically significant relationships between tip placement of subclavian CVCs and BMI (Fig. 3).

**Table 1:**
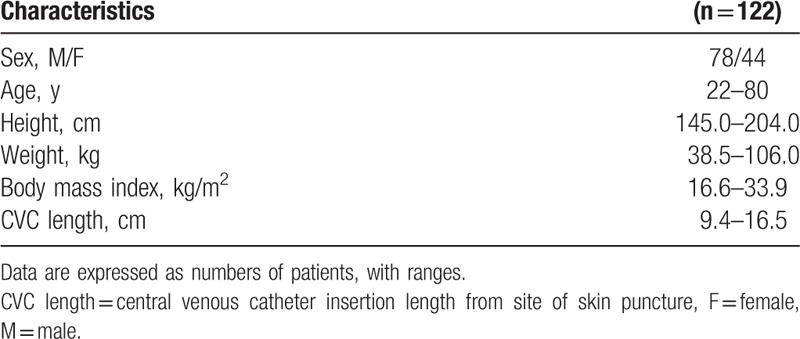
Demographic characteristics of the study patients.

**Table 2:**
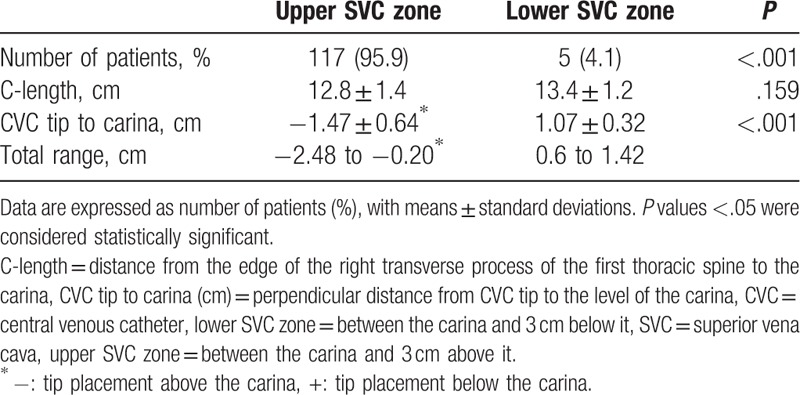
Tip placement of the subclavian CVC using the C-length on a chest radiograph.

**Table 3:**
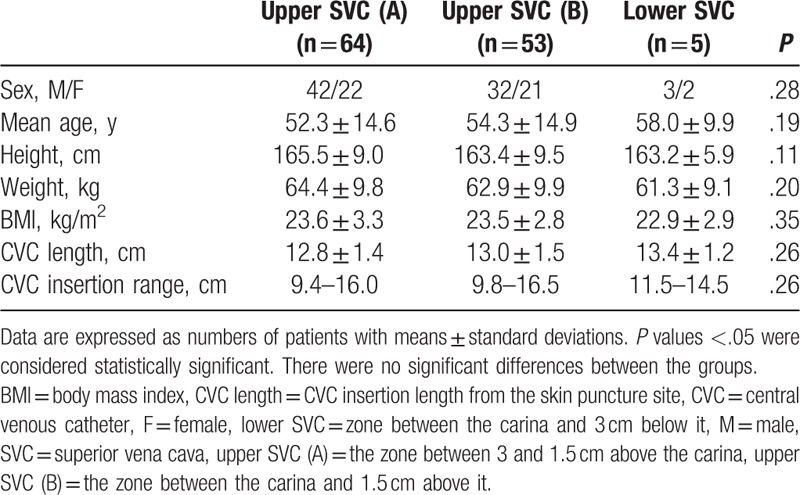
Comparison of patients in whom a subclavian CVC tip was placed in the SVC.

## 4 Discussion

CVCs play an essential role in the care of critically ill patients, but many complications, such as catheter-related sepsis,^[17]^ arrhythmias, and vascular perforation with hydrothorax or cardiac tamponade,^[18]^ can occur; cardiac tamponade in particular is considered one of the most serious complications in the care of critically ill patients.^[10]^ The arrhythmias and perforation observed with tamponade typically result from a malpositioned catheter and thus are potentially preventable.^[11,19]^ To minimize the risk of cardiac tamponade, it has been suggested that the tips of CVCs should be located above the cephalic limit of the pericardial reflection, not merely above the SVC/RA junction.^[15,16,18]^ Use of right atrial ECG or formulae based on patient height can reduce the incidence of malposition of the tip of the CVC,^[20,21]^ but more proximal placement of multilumen CVCs increases the risk of extravasation from the proximal port.^[13]^ Chest radiographs are usually used to confirm the proper positioning of a CVC, with the carina being a reliable landmark for the placement of its tip,^[9–11]^ because the carina is easily visible even in poor-quality portable chest radiographs and is located in the mid-zone of the SVC.^[9]^

Subclavian central venous catheterization is a common procedure, but there is no gold standard for predicting the optimal length of catheter insertion.^[7–9,13]^ Using conventional techniques, it is difficult to place CVC tips above the RA in all cases.^[13]^ Some studies have suggested that a particular fixed length would be an optimal catheter insertion length during right subclavian venous cannulation.^[22,23]^ However, a particular fixed length with a wider range than 7 cm has the risk of misplacement of the tip of the CVC in SVCs with a length of 6 cm,^[1]^ and CVC insertion to a particular fixed depth is not desirable.^[12]^

In our present study, all tips of subclavian CVCs inserted using the C-length were placed in the SVC, and no subclavian CVC entered the RA, because all subclavian CVCs were inserted into their respective C-lengths. This success is partially due to the fact that the target of the C-length was not a point but rather a zone of the SVC. In a previous study, the C-length was determined by measuring the distance between 2 landmarks on preoperative posteroanterior chest radiographs using a PACS, and the individual C-lengths varied widely, even in patients of the same height.^[12]^ We found that there were no significant differences in demographic characteristics among our patients who had CVC insertion in the upper SVC (A), upper SVC (B), or lower SVC. The absence of a significant correlation between height and length of CVC insertion suggests that height alone is not a factor for CVC placement in the SVC, confirming previous findings of no relationship between optimal CVC insertion length and body height or sex.^[10,12]^

There are some limitations to the present study. First, this study was a retrospective study. Second, the C-length method may not be applied under some circumstances such as altered local anatomy (e.g., chest-wall deformity, kyphoscoliosis, carina deviation, vascular injury, prior surgery, radiation history). Finally, the limitation of the C-length method is that it may not be applied to children.

Our present findings indicate that an individually premeasured C-length created using 2 landmarks, namely, the edge of the right transverse process of the first T1 and the carina on preoperative posteroanterior chest radiographs, can be used as an available insertion length for right SCV catheterization to place the tip of the CVC in the SVC in adults. Additional prospective studies are recommended to determine the predictive values of this landmark-guided technique for CVCs placed through the right subclavian vein.
